# Complete Genome Sequence of the Porcine Strain *Brachyspira pilosicoli* P43/6/78^T^

**DOI:** 10.1128/genomeA.00215-12

**Published:** 2013-02-21

**Authors:** Changyou Lin, Henk C. den Bakker, Haruo Suzuki, Tristan Lefébure, Lalit Ponnala, Qi Sun, Michael J. Stanhope, Martin Wiedmann, Gérald E. Duhamel

**Affiliations:** a Department of Biomedical Sciences, College of Veterinary Medicine, Cornell University, Ithaca, New York, USA; b Department of Population Medicine and Diagnostic Sciences, College of Veterinary Medicine, Cornell University, Ithaca, New York, USA; c Department of Food Science, Cornell University, Ithaca, New York, USA; d Biotechnology Center, Cornell University, Ithaca, New York, USA; e Life Sciences Core Laboratories Center, Cornell University, Ithaca, New York, USA

## Abstract

Reported herein is the complete genome sequence of *Brachyspira pilosicoli* strain P43/6/78^T^, isolated from a pig with clinical disease. This sequence will aid in the study of genome-wide comparison among *Brachyspira* species.

## GENOME ANNOUNCEMENT

Among the *Brachyspira* species, *Brachyspira pilosicoli* has the unique capacity to colonize and infect a wide range of hosts, including human beings, nonhuman primates, dogs, pigs, and wild and domestic avian species (1–7). In all hosts, *B. pilosicoli* causes colonic spirochetosis (CS), a polymicrobial disease characterized by persistent intimate mucosal epithelial cell attachment of spirochetes alone or together with certain enterohepatic *Helicobacter* species (4, 8). In developing countries, *B. pilosicoli* can be recovered from stool specimens obtained from immunocompetent individuals, while in Western societies it is found primarily among homosexual men and HIV-positive individuals, some with diarrhea, abdominal pain, and rectal bleeding (9). Systemic spread of *B. pilosicoli* has been reported mostly in critically ill human patients, some with diarrheal disease (10, 11).

In order to further investigate the pathogenesis of CS, the complete or partial genome sequences of several *Brachyspira* species have been determined; however, to date the complete genome sequence of the *B. pilosicoli* porcine isolate P43/6/78^T^ has not been determined or analyzed (1, 3).

The genome sequence of *B. pilosicoli* strain P43/6/78^T^ was determined by using paired-end and shotgun libraries of purified genomic DNA and the 454 pyrosequencing technology (GS FLX titanium genome sequencer system; 454 Life Sciences Corporation, Branford, CT). The total 454 GS FLX reads from both paired-end and shotgun libraries provided approximately 38-fold coverage of the bacterial genome. A combination assembly of the two libraries was done by using Newbler Assembler version 2.0.00.20 (454 Life Sciences Corp.), and 19 scaffolds (>500 bp) were generated. Primer 3 (12) was used to design PCR primers to amplify gaps between scaffolds, and PCR products were sequenced by the Sanger DNA sequencing method at the Cornell University Life Sciences Core Laboratories Center. Final closure of the genome was accomplished by repeated cycles of sequence assembly incorporating gap sequences by using Sequencher version 4.8 (Gene Codes Corporation, Ann Arbor, MI). Genome annotation was performed by the National Center for Biotechnology Information (NCBI) Prokaryotic Genome Automatic Annotation Pipeline.

The genome of *B. pilosicoli* strain P43/6/78^T^ consists of a single circular 2,555,556-bp chromosome with a G+C content of 27.9 mol%. The annotated genome counts 2,208 protein-coding genes, of which 688 represent proteins without a known function (“hypothetical genes”) and 100 genes have been disrupted by a potential frameshift. The type strain of *B. pilosicoli* seems most similar to the previously sequenced porcine strain *B. pilosicoli* 95/1000 (GenBank accession no. CP002025) from Australia (13), with 98.7% average BLAST nucleotide identity and over 93% of the genome that could be aligned to the 95/1000 genome sequence. The two strains show a high level of synteny and share approximately 1,995 orthologous genes. The majority of the genes that are found only in *B. pilosicoli* P43/6/78^T^ but not in 95/1000 are hypothetical genes, but some, such as a beta-galactosidase gene and a gene cluster putatively involved in O-antigen variation, may have important phenotypic implications.

### Nucleotide sequence accession number. 

The complete genome sequence of *B. pilosicoli* strain P43/6/78^T^ has been deposited in GenBank under the accession number CP002873.
